# Titanium Dioxide Nanoparticles Increase Sensitivity in the Next Generation of the Water Flea *Daphnia magna*

**DOI:** 10.1371/journal.pone.0048956

**Published:** 2012-11-07

**Authors:** Mirco Bundschuh, Frank Seitz, Ricki R. Rosenfeldt, Ralf Schulz

**Affiliations:** Institute for Environmental Sciences, University of Koblenz-Landau, Landau, Germany; Argonne National Laboratory, United States of America

## Abstract

The nanoparticle industry is expected to become a trillion dollar business in the near future. Therefore, the unintentional introduction of nanoparticles into the environment is increasingly likely. However, currently applied risk-assessment practices require further adaptation to accommodate the intrinsic nature of engineered nanoparticles. Combining a chronic flow-through exposure system with subsequent acute toxicity tests for the standard test organism *Daphnia magna*, we found that juvenile offspring of adults that were previously exposed to titanium dioxide nanoparticles exhibit a significantly increased sensitivity to titanium dioxide nanoparticles compared with the offspring of unexposed adults, as displayed by lower 96 h-EC_50_ values. This observation is particularly remarkable because adults exhibited no differences among treatments in terms of typically assessed endpoints, such as sensitivity, number of offspring, or energy reserves. Hence, the present study suggests that ecotoxicological research requires further development to include the assessment of the environmental risks of nanoparticles for the next and hence not directly exposed generation, which is currently not included in standard test protocols.

## Introduction

More than 1,000 products, including sunscreens, textiles, and self-cleaning surfaces, either contain engineered nanoparticles or are produced by nanotechnology. As a result, the unintentional introduction of engineered nanoparticles into the environment is increasing [1]. However, potential environmental risks and effects elicited by such nanoparticles on the integrity of ecosystems remain largely unknown [2]. This situation has arisen due to the lack of information regarding current environmental concentrations of nanoparticles, which is primarily caused by the absence of suitable analytical techniques [3]. Models predicting environmental concentrations of nanoparticles are compromised for this reason. A recent study reported a median titanium dioxide nanoparticle (nTiO_2_) concentration within surface waters in the ng to low µg/L range [4]. Moreover, the test protocols currently utilised for environmental risk assessment of nanoparticles were developed to accommodate traditional chemicals, such as pesticides [5]. As nanoparticles exhibit distinct physical and chemical properties, they often present different behaviours relative to their bulk phases and other chemical stressors [6], [7]. Their rapid aggregation, which causes an accumulation at the bottom of the experimental test units, could adversely affect benthic aquatic organisms [8]. At the same time, their bioavailability in the aqueous phase in ecotoxicological experiments is reduced [9]. These unique properties and associated experimental challenges require modifications of the respective test protocols [5].

**Figure 1:**
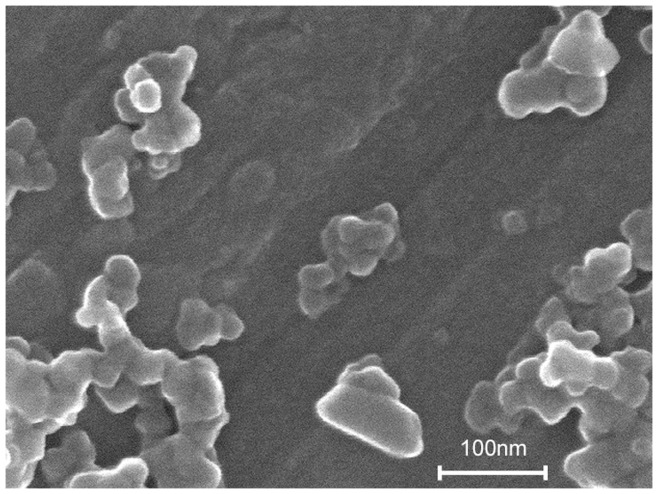
Scanning electron microscope image. An image of the size-homogenised, stable nTiO_2_ suspension of the product P25 taken by an scanning electron microscope using 100,000-fold magnification (Hitachi SU8030).

**Figure 2:**
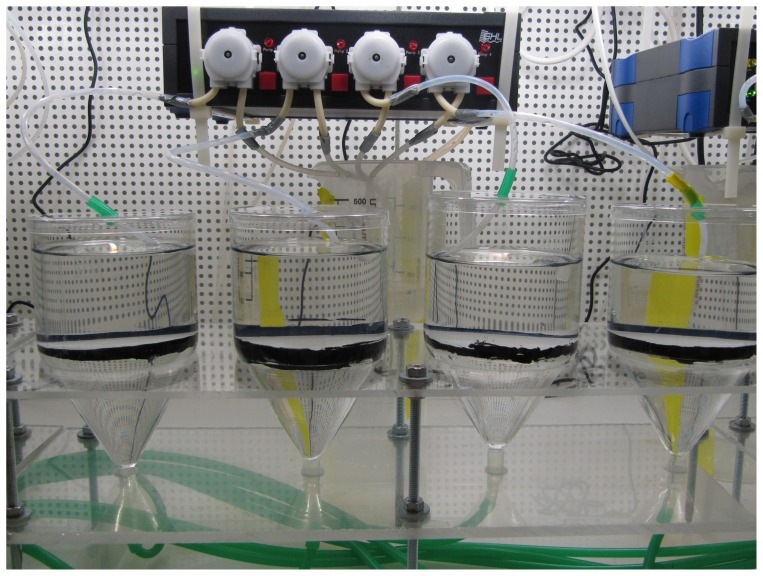
The experimental set-up. The flow-through testing apparatus, showing four experimental units (volume, 500 mL) with five *D. magna* each. Approximately 40 mL of the test medium was introduced every other hour slightly below the surface of the water. The old test medium was passively discharged using hydrostatic pressure at the bottom of each vessel. A fine mesh screen (0.1 mm) prevented the loss of recently hatched juvenile daphnids.

Here, we investigated the chronic adverse effects of nTiO_2_ on the reproduction of *Daphnia magna*, whose parthenogenetic reproduction cycle is for instance described in detail by Zaffagnini [10], by applying two commercially available products with differing crystalline structure, namely P25 (Evonik, Germany; Figure 1) and A-100 (Crenox, Germany), as an additive-free, size-homogenised, stable suspension. The experimental procedure followed largely the standard test protocol designated by the Organisation for Economic Co-operation and Development (OECD) [11]. However, the experiments were performed in a flow-though system avoiding the accumulation of nTiO_2_ aggregates at the bottom of the test vessels as a potential source of ecotoxicological effects (Figure 2) [8]. To allow for inferences on potential effects on the next ( = filial) generation, the fifth brood released by the exposed adults was assessed separately for each treatment (0.00, 0.02, 2.00 mg/L) regarding its acute sensitivity to nTiO_2_ following the respective OECD test protocol [12]. This pathway of effect may be considered as relevant in the context of the present study since similar observations were reported for other chemical stressors such as algae toxins and polyfluorinated substances [13], [14] but also silver nanoparticles [15].

## Materials and Methods

### General Study Design

**Figure 3:**
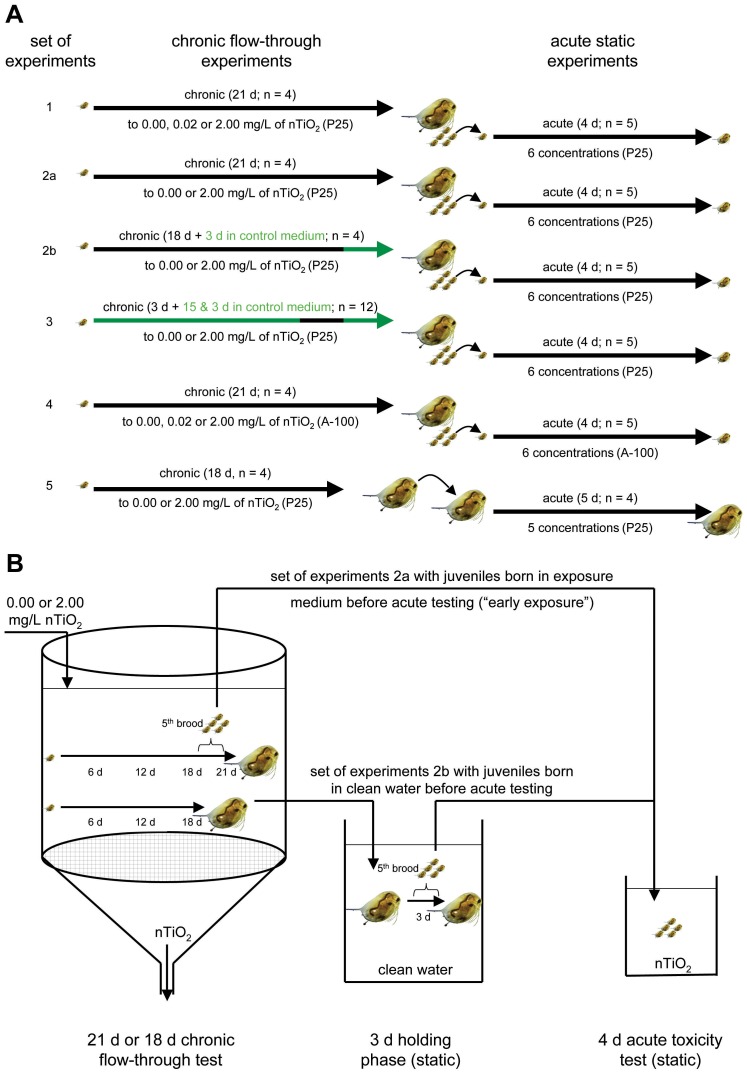
Experimental design. (*A*) Schematic diagram illustrating the experimental procedure of each of the five sets of experiments conducted. (*B*) Visualised experimental procedure for the assessment of the “early exposure” hypothesis (second set of experiments).

In total, five sets of experiments were performed to assess potential effects of nTiO_2_ on the next generation of exposed adult *D. magna* (Figure 3A). During the first set of experiments carried out once, juveniles of the fifth brood released by adult daphnids exposed to 0.00, 0.02 or 2.00 mg/L P25-nTiO_2_ for 21 days were introduced into acute toxicity experiments. The second set of experiments was performed three times to assess the importance of an “early exposure” of juveniles towards nTiO_2_ directly after their release (Figure 3B). Therefore, half of the adults of each treatment, including the control, were transferred to test medium not containing P25-nTiO_2_ approximately 23 hours subsequent to the release of the fourth brood, i.e. after approximately 18 days. Thus, the exposure of newly born juveniles to nanoparticles prior to the initiation of the acute toxicity experiments was avoided. The remaining adults, in contrast, released their juveniles in the respective nTiO_2_ treatment (as in the first set of experiments). The third set of experiments – performed once – intended to investigate implications on juveniles’ sensitivity potentially driven by the exposure of daphnid’s eggs towards nTiO_2_ within the brood pouch. Therefore, adult daphnids were exposed to 0.00 and 2.00 mg/L nTiO_2_ starting with the release of the third brood and lasting until 23 h after the release of the fourth brood resulting in an exposure period of approximately 3 days. This procedure limited any chronic implications potentially transferred from adults to juveniles and represented the maximum time period eggs may have been exposed to nTiO_2_ during earlier experiments. Subsequently, adults were transferred to clean medium for the release of the fifth brood. To investigate whether the results obtained with P25 may be transferable to other nTiO_2_ forms, the experimental procedure used for the first set of experiments was carried out with the product A-100 during the fourth set of experiments. The fifth set is not further described in the manuscript. It was utilized to assess the sensitivity of adult daphnids following the 18 days of exposure to 0.00 and 2.00 mg/L nTiO_2_ (P25), which is displayed in Figure S1.

### Organisms

*D. magna* (Clone V, Eurofins-GAB laboratories, Germany) were cultured at 20±1°C with a 16:8 hour (light:dark) photoperiod in ASTM reconstituted with hard freshwater that was enriched with selenium, vitamins (thiamine hydrochloride, cyanocobalamine, biotine) and seaweed extract (Marinure®, Glenside, Scotland). The daphnids were fed with the green algae *Desmodesmus* sp. on a daily basis (∼200 µg carbon per organism).

### Flow-through Tests

The experimental procedure followed the standard test protocol designated by the OECD [11], with minor deviations. Briefly, each replicate consisted of 500 mL test medium and five *D. magna* that were younger than 24 hours of age at the start of the experiments. The study duration was set at 21 days, and the offspring were counted daily as a measure of the sublethal effect. Most importantly, the experiments were performed in a flow-though system. Therefore, the ASTM medium (148 mL) was mixed with the nTiO_2_ stock solution (12 mL) immediately before delivery in equal proportions to four independent replicates, each with a volume of 500 mL. The old test medium was passively discharged through a mesh at the bottom of the vessel (Figure 1). This procedure was repeated every other hour, ensuring a complete water exchange within 24 hours. The ASTM medium was renewed daily and amended in an age-dependent manner with food (*Desmodesmus* sp.; 50–100 µg carbon/test organism). The nTiO_2_ stock solutions were renewed at 72 h intervals. All of the pumps were equipped as completely as possible with Teflon tubes to minimise the loss of nTiO_2_ during pumping. The flow-through apparatus avoided the accumulation of nTiO_2_ aggregates at the bottom of the test vessels, eliminating one potential source of ecotoxicological effects [8]. Moreover, the medium was amended with seaweed extract to simulate dissolved organic matter that is normally present in natural water, which stabilised nTiO_2_ within the aqueous phase [16]. This procedure ensured a continuous exposure to nTiO_2_ particles of sizes <150 nm (Table S1). The average particle size was monitored daily during the chronic experiments for the 2.00 mg/L nTiO_2_ treatment. Moreover, the actual zeta potential of both products in the test medium was analysed. Because even the highest test concentration delivered in this study was still too low to measure the zeta potential, the respective nTiO_2_ stock suspension was diluted in test medium prior to measurement (Table S1).

### Acute Toxicity Experiments

The fifth brood released by the exposed adults was assessed separately for each treatment regarding its sensitivity to nTiO_2_. The concentration of nTiO_2_ that resulted in 50% immobility of the juvenile daphnids after 96 hours of exposure (96 h-EC_50_) was used as the measure of sensitivity. Because this prolonged study duration was recently recommended for nanoparticle testing [9], the OECD test protocol for acute toxicity tests with *D. magna* [12] was adapted correspondingly, and the daphnids were exposed to 0.00, 0.50, 1.00, 2.00, 4.00 or 8.00 mg/L nTiO_2_.

### Nanoparticle Characterisation

Both of the nTiO_2_ products used in this study, P25 (Evonik) and A-100 (Crenox), were purchased as powdered reagents. Subsequently, both products were prepared as dispersant- and additive-free, size-homogenised, stable suspensions by stirred media milling [9]. The zeta potential and the actual particle size distribution of both suspensions were determined *via* electrophoretic mobility and dynamic light scattering (Delsa™ Nano C, Beckman Coulter, Germany), respectively. The concentrations of nTiO_2_ in the 2.00 mg/L treatment were verified weekly by inductively coupled plasma mass spectrometry [9]. These analyses were supplemented by scanning electron microscope (Hitachi SU8030) imaging for P25 for the verification of particle size.

### Statistical Analysis

Immobilisation data gained from the acute toxicity tests were adjusted by Abbott’s formula if necessary and fitted to adequate dose-response models – based on Akaike Information Criterion and expert judgement – in order to determine 96 h-EC_50_ values using the drc extension package [17] for the statistics program R version 2.13.0. Confidence interval (CI) testing was accomplished to assess for statistically significant differences between 96 h-EC_50_ values of juveniles released by daphnids exposed to the control and those exposed to nTiO_2_ obtained during the first, third, fourth and fifth set of experiments [18]. The 96 h-EC_50_ values of the first and second set of experiments were combined in a meta-analysis assessing for difference between juveniles of the fifth brood released by daphnids not exposed to nTiO_2_ and those exposed to either 0.02 or 2.00 mg/L P25-nTiO_2_. Therefore, a fixed effect model based on the standardised effect size Cohen’s *d* was applied. An effect size was considered to be statistically significant when the respective CI did not include the zero value. Additionally, the respective *p*-values were computed [19].

## Results

### First and Second Set of Experiments

**Figure 4:**
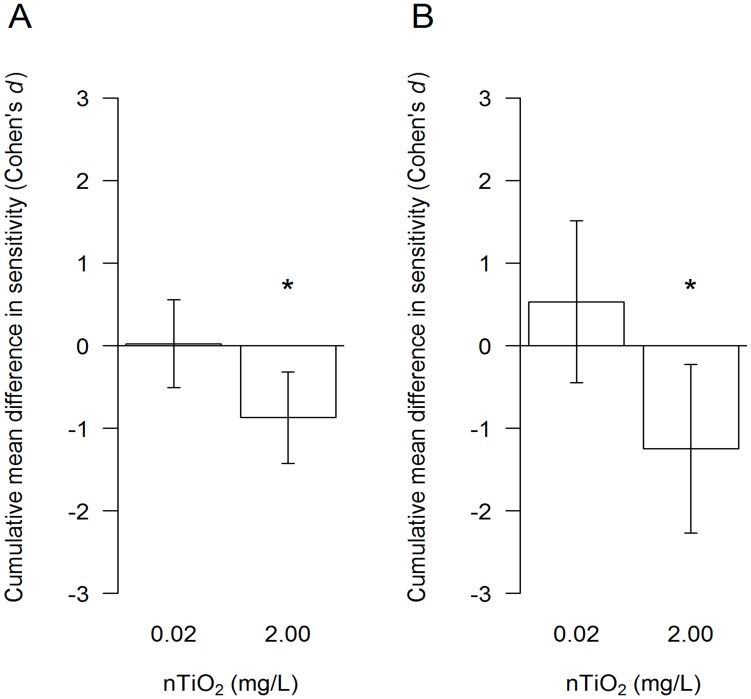
The sensitivity of juveniles released by nTiO_2_-exposed adults. The cumulative mean (±95% CIs) difference in sensitivity – in terms of 96 h-EC50 values – of the offspring (fifth brood) released by adults exposed to 0.02 or 2.00 mg nTiO2/L and offspring released by control (uncontaminated) daphnids is displayed using the standardised effect size Cohen’s *d*. (*A*) The cumulative effect sizes for all bioassays conducted (n = 7) with the fifth brood during the first and second set of experiments. (*B*) The cumulative effect sizes for acute toxicity experiments conducted with offspring released in the control medium by adults previously exposed to the above-mentioned nTiO2 concentrations during the second set of experiments (n = 3). The statistical significance of a cumulative effect is highlighted by an asterisk (*). Negative effect sizes indicate increased toxicity.

The P25-nTiO_2_ particles were dispersed within the aqueous phase throughout the whole chronic study duration and displayed a mean size of 135.8 nm, with approximately 30% of the particles <100 nm (Table S1). The nanoparticles did not adversely affect the mean number of offspring released by exposed adults (Figure S2 left). However, subsequent acute toxicity studies (first set of experiments) showed for the fifth brood released by adults exposed to 2.00 mg/L P25-nTiO_2_ a significantly lower 96 h-EC_50_ value (difference between 96 h-EC_50_ values: 4.39 mg/L, 95% CI of the difference 0.62 to 8.15; Figure S3). Additionally, a meta-analysis, which considered all 96 h-EC_50_ values of the first and the second set of experiments, supported these results (p = 0.0021 with n = 7; Figure 4A). This holds also true for a second meta-analysis that accounted exclusively for the effect sizes of the offspring that originated from the adults exposed to nTiO_2_ but that were released into nanoparticle-free test medium, which hence avoided the possibility of an early exposure (p = 0.0169 with n = 3; Figure 4B).

### Third Set of Experiments

**Figure 5:**
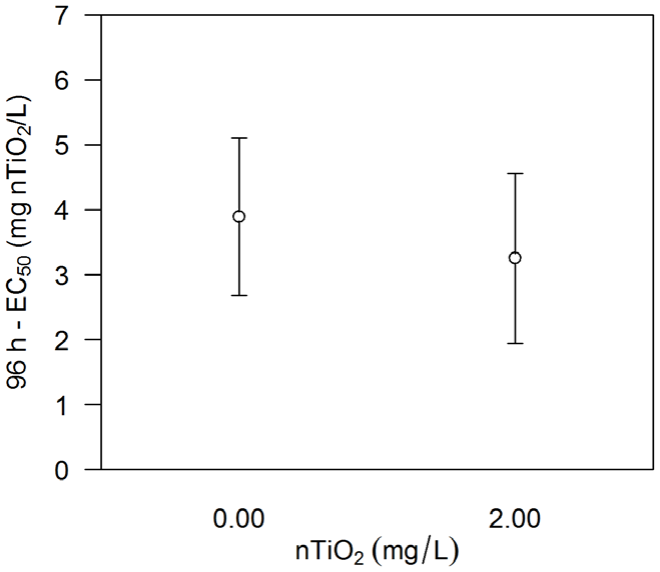
The sensitivity of juveniles released during the third set of experiments. 96 h- EC_50_ values with respective 95% CIs of the fifth brood released by adults exposed to 0.00 and 2.00 mg/L P25-nTiO_2_ during the third set of experiments, which considered exclusively potential implication of nTiO_2_ exposure within the brood pouch. No statistically significant difference among treatments was observed.

No difference in the sensitivity of the offspring (fifth brood) released by adults exposed for approximately 3 days to 0.00 and 2.00 mg/L nTiO_2_ was observed (the difference between 96 h-EC_50_ values: 0.64 mg/L, 95% CI of the difference −1.04 to 2.32; Figure 5).

### Fourth Set of Experiments

**Figure 6:**
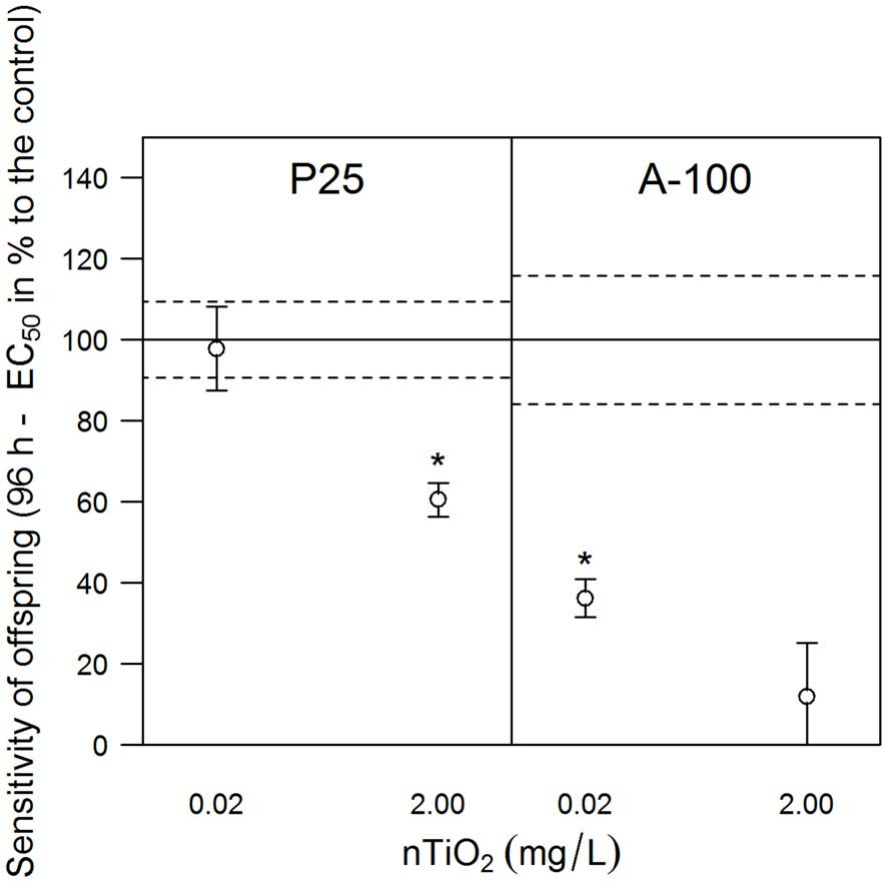
The sensitivity of juveniles released by adults exposed to different nTiO_2_ products. Sensitivity, displayed as percent relative to the 96 h-EC_50_ of the respective control, of the fifth brood released by adult *D. magna* exposed to different nTiO_2_ treatments using the products P25 or A-100. The data displayed for P25 represent the weighted mean values of the seven experiments (first and second set of experiments), each with four replicates per treatment, whereas the 96 h-EC_50_ for the offspring released from the control parents was 3.13 mg/L nTiO_2_. For the product A-100, the results of one experiment with four replicates of pre-treatment are displayed (fourth set of experiments). In this situation, the 96 h-EC_50_ for the offspring released from the control parents was 1.98 mg/L nTiO_2_. The error bars and dashed lines indicate the standard error. The dashed lines are related to the control. Asterisks (*) denote significant differences between a treatment and the respective control.

The chronic exposure to A-100 (for particle characteristics see Table S1) did not affect the reproduction of the test species *D. magna,* at concentrations up to 2.00 mg/L (Figure S2, right). Nevertheless, the offspring released by adults exposed to 0.02 mg/L A-100 were significantly more sensitive than those released by adults from the nanoparticle free control (difference between 96 h-EC_50_ values: 1.26 mg/L, 95% CI of the difference 0.62 to 1.90; Figure 6, right). The sensitivity of the offspring released by adults exposed to high nTiO_2_ concentrations was increased by a factor of at least five. However, it was not possible to verify this deviation by statistical analysis due to the lack of suitable quantitative approaches (Figure 6, right).

## Discussion

The first set of experiments showed that the fifth brood released by adults exposed to 2.00 mg/L P25-nTiO_2_ was significantly more sensitive than the juvenile offspring released by the non-exposed control adults (Figure S3), indicating the possibility of effects passed from the parental to the filial generation of daphnids. However, these results might also be explained by the exposure of the offspring immediately after hatching and prior to their introduction into the acute toxicity tests (Figure 3A). In contrast, the exposure of the juveniles from the nanoparticle-free control commenced up to 24 hours later, with the start of the acute toxicity experiments. To test this “early exposure” possibility, we performed further *D. magna* reproduction assays. However, half of the adults for each treatment during the second set of experiments (Figure 3B), including the control adults, were transferred to test medium containing no nTiO_2_ approximately 23 hours subsequent to the release of the fourth brood. The exposure of the newly released fifth brood to nanoparticles prior to the initiation of the acute toxicity experiments was thus avoided. In contrast, the remaining adults released their offspring into the respective treatment conditions. Three additional independent experiments were performed using this experimental set-up. All of the 96 h-EC_50_ (n = 7) values obtained for the offspring released by adults exposed to nTiO_2_ were combined into a meta-analysis (Figure 4A), which was based on a fixed effect model using Cohen’s *d* as a standardised effect size. Furthermore, a second meta-analysis was performed considering exclusively the effect sizes of the offspring that originated from the adults exposed to nTiO_2_ but that were released into nanoparticle-free test medium (n = 3; Figure 4B). Both meta-analyses revealed a significant increase in the sensitivity of the offspring released by adults exposed to 2.00 mg/L nTiO_2_ compared to offspring released from adults not suffering from nTiO_2_ exposure. These results suggest that “early exposure” is insufficient to explain the approximately twofold increase in sensitivity (Figure 6, left) observed in the fifth brood released from the exposed adults. However, the daphnids’ eggs might already be exposed to nTiO_2_ within the brood pouch, which could potentially affect the sensitivity of the subsequent offspring [20]. To assess this issue, adult daphnids were exposed to 0.00 and 2.00 mg/L nTiO_2_ starting with the release of the third brood and lasting until at least 23 h after the release of their fourth brood (third set of experiments). This procedure alleviated any long-term implications potentially transferred from adults to offspring and represented the maximum time period over which eggs may have been exposed to nTiO_2_ during our earlier experiments. Subsequently, the adults were transferred to nTiO_2_-free medium for the release of the fifth brood. Acute toxicity experiments revealed no difference in the sensitivity of the offspring released by adults exposed to 0.00 and 2.00 mg/L nTiO_2_ (Figure 5), indicating that the exposure of the eggs during the early phases of development could not account for the observed effects. Consistent with another study [15], which uncovered adverse effects of silver nanoparticles ingested by adult fruit fly *Drosophila melanogaster* (Meigen) passed on to their offspring, a similar explanation might apply for this study. Comparable observations were also reported for other chemical stressors like algal toxins [13], perfluorooctane sulfonic acid, perfluorooctanoic acid [14], vinclozolin and 5-azacytidine [21]. However, in this study, we have uncovered effects induced by nTiO_2_ on the next generation of *D. magna* but not the parental generation, in terms of quantitative ecotoxicological endpoints: sensitivity to nTiO_2_ after 18 days of exposure, number of offspring, and lipid content (Figure S1; S2, left; S4), while the mechanism resulting in this observation have no yet been understood.

Although this study demonstrates, for the first time, the possibility of nTiO_2_ effects on the next generation of *D. magna* in which the parental generation exhibits no obvious effects, whether these effects would also result from exposure to other TiO_2_ products remains unclear. Therefore, another flow-through experiment followed by acute toxicity experiments was performed with the product A-100 (fourth set of experiments; Figure 3A). Exposure to A-100 also did not affect the reproduction of the test species *D. magna,* measured as the number of offspring released at a product concentration of up to 2.00 mg/L (Figure S2, right). Dabrunz et al. [9] reported a 96 h-EC_50_ of 0.74 mg/L for this product. This deviation in effects may be explained by the amendment of the test medium with seaweed extract [16] as well as by the avoidance of nTiO_2_ deposition on the bottom of the test vessels [8]. Nonetheless, the offspring released by adults that were exposed to even 0.02 mg/L A-100 were significantly more sensitive–by an approximate factor of 3–than those released by the control parents (Figure 6, right). Moreover, the sensitivity of the offspring released by adults exposed to high nTiO_2_ concentrations was increased by a factor of at least five (Figure 6, right). These results might represent general safety implications of nTiO_2_ products, although their intensity varies among products and the concentration applied.

### Conclusion

Although the test design of this study ensured the bioavailability of the investigated nanoparticles over the whole study duration, the parental *D. magna* did not exhibit any indications of toxic stress under these standard test conditions. However, the offspring generation of daphnids released by parental *Daphnia* that were previously exposed to nTiO_2_ were significantly more sensitive to nTiO_2_. Finally, the present study suggests that the standardised testing protocols that comprise the foundation of the current environmental risk-assessment approaches for nanoparticles underestimate risks and require modification. Apart from the recently recommended extension of the study duration for acute toxicity testing [9], the OECD test guidelines for the assessment of chronic ecotoxicity need to be improved by considering the effects on the next generation, even on offspring that has never been directly exposed to the agents.

## Supporting Information

Figure S1 120 h- EC_50_ values with respective 95% CIs of adult *D. magna* following 18 days of exposure to nTiO_2_ (P25) in the flow-through system (fifth set of experiments). No statistically significant deviations regarding the sensitivity were detected.
(PDF)

Figure S2 Boxplot (bold line represents the median) of the offspring per test organism (n = 20) exposed to P25 (first set of experiments) or A-100 (fourth set of experiments) nTiO_2_ after 21 days of exposure to 0.00, 0.02 or 2.00 mg nTiO_2_/L.
(PDF)

Figure S3 96 h-EC_50_ values with respective 95% CIs of the fifth brood released by adults exposed to P25 nTiO_2_ during the flow-through experiment (first set of experiments); Asterisk (*) denotes statistically significant difference between the juveniles released from adults exposed to 2.00 mg/L TiO_2_ and the control based on confidence interval testing (difference between 96 h-EC_50_ values 4.39 mg/L, 95% CI 0.62 to 8.15).
(PDF)

Figure S4 Lipid content per adult *D. magna* after 21 days of exposure to P25 nTiO_2_ (first set of experiments).
(PDF)

Table S1 Particle characteristics of P25 and A-100: The table displays the 10^th^ and the 90^th^ percentile of the particle size distribution, the mean percentage of particles below a particle size of 100 nm as well as the mean particle size together with the polydispersity index. This index provides information on the range of the particle size distribution. A value above 0.3 indicates unreliability of the measurement, due to masking of small particles by large ones. Additionally the zeta potential of the particles and their measured concentration in the test medium is given. nTiO_2_ concentrations were measured following Dabrunz et al.
(PDF)
