# Supplementary material for: Titanium Dioxide Nanoparticles Increase Sensitivity in the Next Generation of the Water Flea Daphnia magna
Source: PLoS One. 2012 Nov 7;7(11):e48956. doi: 10.1371/journal.pone.0048956 (PMC3492132; doi:10.1371/journal.pone.0048956)

Supplementary Information Figure S1. 120 h- EC_{50} values with respective 95% CIs of adult *D. magna* following 18 days of exposure to $nTiO_2$ (P25) in the flow-through system (fifth set of experiments). No statistically significant deviations regarding the sensitivity were detected.

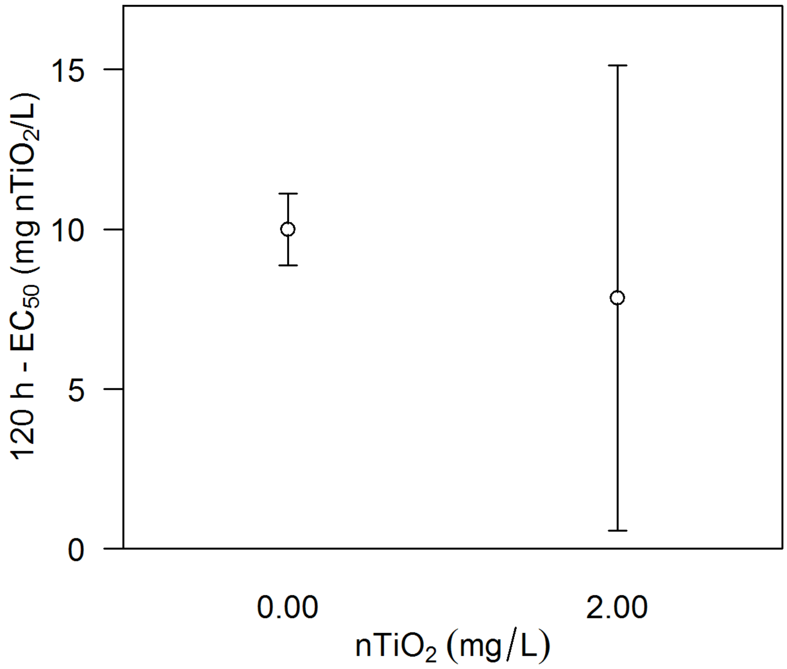

Supplement: Figure S1 — 120 h- EC50 values with respective 95% CIs of adult D. magna following 18 days of exposure to nTiO2 (P25) in the flow-through system (fifth set of experiments). No statistically significant deviations regarding the sensitivity were detected. (PDF) [file pone.0048956.s001.pdf]
