# Supplementary material for: Titanium Dioxide Nanoparticles Increase Sensitivity in the Next Generation of the Water Flea Daphnia magna
Source: PLoS One. 2012 Nov 7;7(11):e48956. doi: 10.1371/journal.pone.0048956 (PMC3492132; doi:10.1371/journal.pone.0048956)
Supplement: Figure S2 — Boxplot (bold line represents the median) of the offspring per test organism (n = 20) exposed to P25 (first set of experiments) or A-100 (fourth set of experiments) nTiO2 after 21 days of exposure to 0.00, 0.02 or 2.00 mg nTiO2/L. (PDF) [file pone.0048956.s002.pdf]

Supplementary Information Figure S2. Boxplot (bold line represents the median) of the offspring per test organism (n=20) exposed to P25 (first set of experiments) or A-100 (fourth set of experiments) $nTiO_2$ after 21 days of exposure to 0.00, 0.02 or 2.00 mg $nTiO_2$/L.

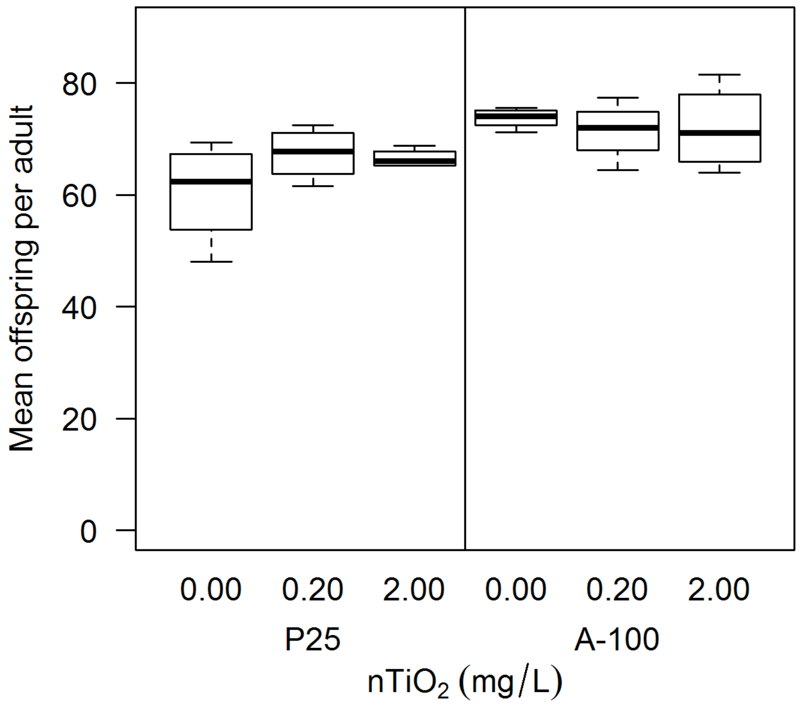

In total, six reproduction experiments were performed. However, in only two of them, i.e. the first and fourth set of experiments, the cumulative offspring was assessed over 21 days (P25, n=4; A-100, n=4). Data were checked for normal distribution (Shapiro-Wilks test) and homogeneity of variances (Bartlett's test). Since the requirements for parametric testing were not met, Kruskal-Wallis Rank Sum Test was utilized to assess statistical significance. The statistical analysis revealed for both experiments no statistical significant difference among treatment groups (P25: p=0.1274; A-100: p=0.1174).
