# Supplementary material for: Titanium Dioxide Nanoparticles Increase Sensitivity in the Next Generation of the Water Flea Daphnia magna
Source: PLoS One. 2012 Nov 7;7(11):e48956. doi: 10.1371/journal.pone.0048956 (PMC3492132; doi:10.1371/journal.pone.0048956)

Supplementary Information Figure S3. 96 h-EC_{50} values with respective 95% CIs of the fifth brood released by adults exposed to P25-nTiO_2 during the flow-through experiment (first set of experiments); Asterisk (*) denotes statistically significant difference between the juveniles released from adults exposed to 2.00 mg/L TiO_2 and the control based on confidence interval testing (difference between 96 h-EC_{50} values 4.39 mg/L, 95% CI 0.62 to 8.15).

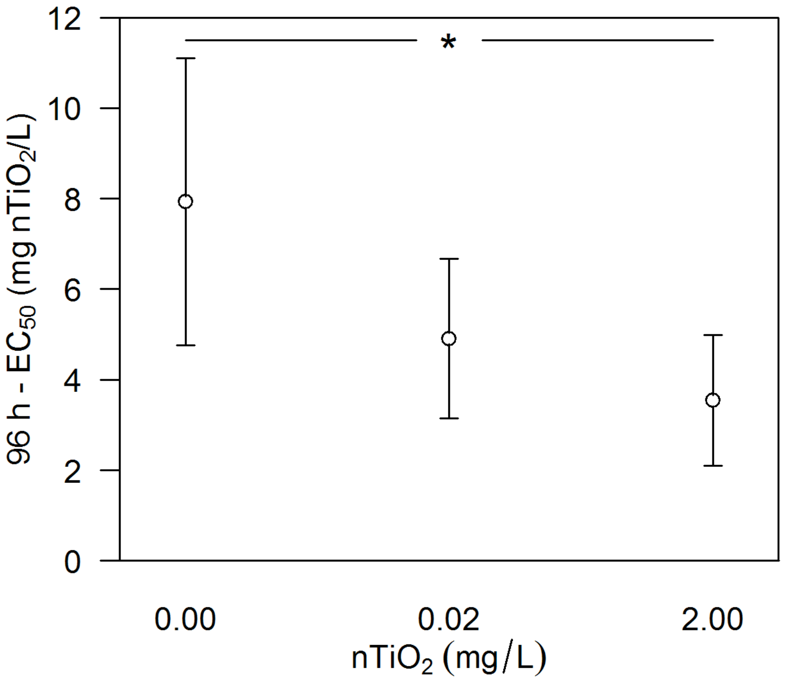

Supplement: Figure S3 — 96 h-EC50 values with respective 95% CIs of the fifth brood released by adults exposed to P25 nTiO2 during the flow-through experiment (first set of experiments); Asterisk (*) denotes statistically significant difference between the juveniles released from adults exposed to 2.00 mg/L TiO2 and the control based on confidence interval testing (difference between 96 h-EC50 values 4.39 mg/L, 95% CI 0.62 to 8.15). (PDF) [file pone.0048956.s003.pdf]
