# Supplementary material for: Titanium Dioxide Nanoparticles Increase Sensitivity in the Next Generation of the Water Flea Daphnia magna
Source: PLoS One. 2012 Nov 7;7(11):e48956. doi: 10.1371/journal.pone.0048956 (PMC3492132; doi:10.1371/journal.pone.0048956)
Supplement: Figure S4 — Lipid content per adult D. magna after 21 days of exposure to P25 nTiO2 (first set of experiments). (PDF) [file pone.0048956.s004.pdf]

Supplementary Information Figure S4. Lipid content per adult *D. magna* after 21 days of exposure to P25-nTiO_2 (first set of experiments).

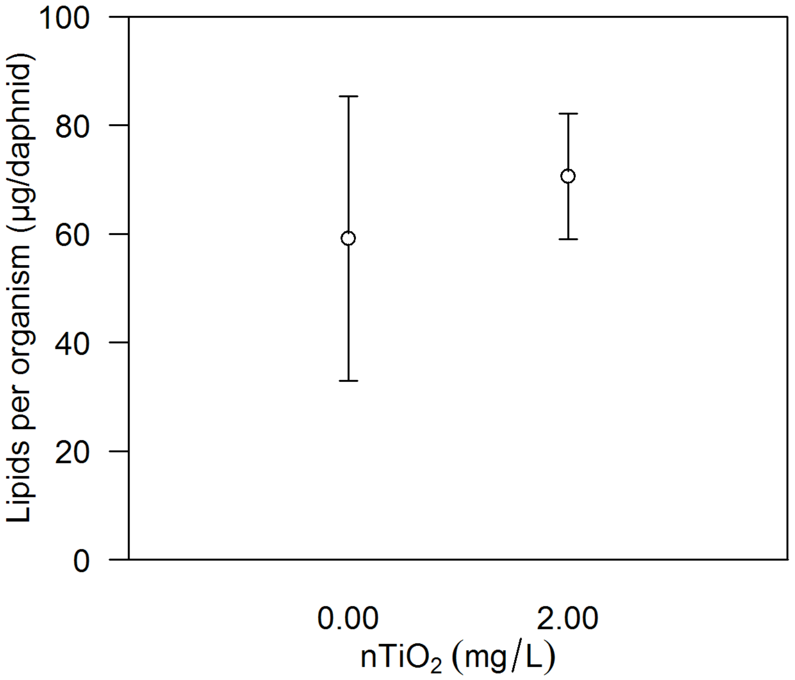

After being exposed for 21 d, animals from the 0.00 and 2.00 mg/L P25 treatment were shock-frozen and freeze-dried for approximately 48 h. Five organisms of each treatment were subjected to a lipid analysis as described in detail by Zubrod et al.[1]. Briefly, test organisms were placed for 72 h in 0.5 mL of a 1:1 chloroform:methanol solution before being ground in glass mortars. Afterwards samples were filtered using a syringe filter (0.1 µm) with a polytetrafluorethylene membrane (Whatman, Germany) to exclude a potential influence of nTiO_2 particles on the measurement. The resulting filtrates were transferred to culture tubes and the solvent was evaporated in a water bath (95°C). Thereafter, 0.2 mL of sulfuric acid (97%) were added followed by 10 min of heating at 95°C. Prior to addition of 5 mL of vanillin reagent, the samples were cooled to room temperature. Subsequently, 60 µL of each sample was transferred to a 96-well microwell plate (TC MicroWell 96F SI W/Lid Nunclon

D, Nunc). The absorbance was measured at 490 nm and the total lipid content of daphnids was quantified using a standard curve prepared with commercially available soybean oil.

Since the data of the lipid analysis did not fulfill the requirements for parametric testing a Wilcoxon rank sum test was accomplished, displaying no statistical differences between the lipid content of daphnids previously exposed to 0.00 and 2.00 mg/L P25 ($p=0.5476$).

Reference

1. Zubrod, J. P., Bundschuh, M., Feckler, A., Englert, D. & Schulz, R. Ecotoxicological impact of the fungicide tebuconazole on an aquatic decomposer-detritivore system. *Environ. Toxicol. Chem.* 30, 2718-2724 (2011).
