# Supplementary material for: Titanium Dioxide Nanoparticles Increase Sensitivity in the Next Generation of the Water Flea Daphnia magna
Source: PLoS One. 2012 Nov 7;7(11):e48956. doi: 10.1371/journal.pone.0048956 (PMC3492132; doi:10.1371/journal.pone.0048956)
Supplement: Table S1 — Particle characteristics of P25 and A-100: The table displays the 10th and the 90th percentile of the particle size distribution, the mean percentage of particles below a particle size of 100 nm as well as the mean particle size together with the polydispersity index. This index provides information on the range of the particle size distribution. A value above 0.3 indicates unreliability of the measurement, due to masking of small particles by large ones. Additionally the zeta potential of the particles and their measured concentration in the test medium is given. nTiO2 concentrations were measured following Dabrunz et al. (PDF) [file pone.0048956.s005.pdf]

Table S1. Particle characteristics of P25 and A-100: The table displays the 10th and the 90th percentile of the particle size distribution, the mean percentage of particles below a particle size of 100 nm as well as the mean particle size together with the polydispersity index. This index provides information on the range of the particle size distribution. A value above 0.3 indicates unreliability of the measurement, due to masking of small particles by large ones. Additionally the zeta potential of the particles and their measured concentration in the test medium is given. $nTiO_2$ concentrations were measured following Dabrunz et al.[1].

product	mean (±SD; n) particle size nm	10th percentile (mean±SD; n) of particle size distribution nm	90th percentile (mean±SD; n) of particle size distribution nm	mean percentage (mean±SD; n) of particles below a size of 100 nm	polydispersity index PI	mean (±SD; n) zeta potential mV	nominal concentration mg/L	mean (±SD; n) measured concentration mg/L		
								1st week	2nd week	3rd week
P25	135.8 (±14.1; 30)	76.2 (±7.4; 30)	251.5 (±57.8; 30)	31.30 (±6.62; 30)	0.181-0.270	-19.46 (±0.41; 3)	0.00	0	0	0
							2.00	1.62 (±0.07; 3)	1.64 (±0.02; 3)	1.90 (±0.03; 3)
A-100	143.2 (±23.9; 22)	74.8 (±6.4; 22)	285.6 (±103.9; 22)	31.89 (±5.01; 22)	0.143-0.284	-16.75 (±0.29; 3)	0.00	0	0	0
							2.00	2.34 (±0.26; 3)	2.39 (±0.17; 3)	2.42 (±0.07; 3)

Reference

1. Dabrunz A, Duester L, Prasse C, Seitz F, Rosenfeldt RR, Schilde R, Schaumann GE, Schulz R (2011) Biological surface coating and molting inhibition as mechanisms of TiO_2 nanoparticle toxicity in *Daphnia magna*. PLoS One 6: e20112.
